# Identification and validation of an endotoxin tolerance–based prognostic model with therapeutic insights in sepsis

**DOI:** 10.3389/fimmu.2025.1707490

**Published:** 2026-01-15

**Authors:** Yu Xie, Yadong Su, Yin Qin, Qiuhong Zhang, Jie Liu, Yanlin Wu, Ning Du, Yu Jiang, Gang Liu

**Affiliations:** 1 Department of Respiratory and Critical Medicine, University-Town Hospital of Chongqing Medical University, Chongqing, China; 2 Department of Emergency and Critical Care Medicine, University-Town Hospital of Chongqing Medical University, Chongqing, China; 3 Center for Mental Health, University-Town Hospital of Chongqing Medical University, Chongqing, China; 4 Department of Emergency and Critical Care Medicine, The Affiliated Yongchuan Hospital of Chongqing Medical University, Chongqing, China

**Keywords:** endotoxin tolerance, immunosuppression, machine learning, prognosis, sepsis

## Abstract

**Background:**

Sepsis outcomes remain difficult to predict because immune trajectories are heterogeneous and dynamically shift from early activation to immunosuppression. Endotoxin tolerance (ET) in circulating monocytes/macrophages is a key mechanism of sepsis-associated immunosuppression but has not been systematically leveraged for prognostication. We sought to develop and clinically validate an ET-related gene (ETG) signature for short-term mortality risk stratification.

**Method:**

Public whole-blood transcriptomic datasets were intersected with curated ET gene sets to derive ETG candidates. An ensemble machine-learning framework (108 model/feature-selection combinations across 12 algorithms) was used to build and rank prognostic models for 28-day mortality; the final parsimonious signature (10 ETGs, including IL4R, ATM, CX3CR1, FCGR1A) informed a risk score. A two-variable nomogram (age + ETG risk score) was constructed. Internal performance was assessed by bootstrapped calibration, time-dependent ROC/AUC at 7, 14, and 28 days, and Harrell’s C-index. Experimental validation used a prospective ICU cohort (n=50; 13 survivors, 37 non-survivors). PBMCs were profiled by RT-qPCR and Western blot; CD14^+^ monocytes were analyzed by flow cytometry for FCGR1A/CD64, CX3CR1, and PD-1. We further explored regulatory context (ceRNA network) and druggability (DGIdb query and molecular docking to CX3CR1, FCGR1A, and TLR5).

**Results:**

The 10-gene ETG signature stratified mortality risk with acceptable discrimination and calibration. AUCs were 0.76 (95% CI 0.69–0.83), 0.78 (0.72–0.83), and 0.73 (0.67–0.78) at 7, 14, and 28 days, respectively; Harrell’s C-index was 0.782. The age-integrated nomogram showed close agreement between predicted and observed survival across timepoints. Functional enrichment indicated immune-response pathways enriched in the low-risk group. In the clinical cohort, non-survivors exhibited lower mRNA and protein levels of FCGR1A, TLR5, and CX3CR1 in PBMCs; flow cytometry revealed reduced proportions of FCGR1A^+^, CX3CR1^+^, and PD-1^+^ CD14^+^ monocytes (p<0.01). The ceRNA analysis highlighted a putative NEAT1/miR-1287-5p/CX3CR1 axis. Docking suggested plausible ligandability of CX3CR1, FCGR1A, and TLR5, nominating candidates such as valproic acid and CGP-52608 for follow-up testing.

**Conclusion:**

An ET-anchored, 10-gene signature captures a clinically relevant axis of sepsis-associated immunosuppression and enables short-term mortality risk stratification. Integration with age yields a simple nomogram with stable internal performance. Multilayer validation (transcript, protein, single-cell) supports biological plausibility. Prospective multicenter studies with richer clinical annotation and functional assays are warranted to confirm generalizability and to evaluate ET-guided immunomodulatory strategies.

## 1 Introduction

Sepsis is characterized by a dysregulated and life-threatening host response to infection, often accompanied by multiple organs failure and death (1–3). Although early fluid resuscitation, timely antibiotics, and organ support have improved care, mortality remains high at roughly 26% (4, 5). The immune course of sepsis is biphasic: an initial surge of inflammation is followed within days to weeks by a sustained state of immunosuppression (6, 7). However, the administration of anti-inflammatory agents in over 30 clinical trials for sepsis treatment has yielded no discernible benefits (8, 9), suggesting a potential need for a paradigm shift in our understanding from hyperinflammation to immunosuppression. Prolonged immunosuppression during sepsis impairs the host’s ability to effectively eliminate pathogens and makes the host more vulnerable to secondary infections. Consequently, this results in increased long-term morbidity and mortality rates (9). Therefore, exploring prognostic signatures in patients with sepsis, while accounting for the role of immunosuppression, is crucial.

Endotoxin tolerance (ET) is a canonical mechanism of sepsis-induced immunosuppression. After prior exposure to lipopolysaccharide (LPS), monocytes and macrophages mount a blunted response to subsequent stimulation, with reduced production of pro-inflammatory cytokines and relative increases in anti-inflammatory mediators (10–14). ET is not simply anti-inflammation; it reflects impaired cellular immune function and “loss” of effective immune memory (13). In septic patients, monocytes show diminished antigen-presenting capacity and down-regulation of CD80, CD40, and HLA-DR after LPS challenge ex vivo (15–17). Clinically, an ET-like phenotype in circulating monocytes correlates with prolonged intensive-care stay, longer mechanical ventilation, and more secondary infections (18). Survivors tend to regain LPS responsiveness, whereas non-survivors often do not (19). ET can persist beyond two weeks even in healthy volunteers given two LPS challenges (20), and has been linked to higher mortality in sepsis (21). Despite this body of work, the prognostic value of ET-related genes (ETGs) has not been comprehensively defined in large, integrated analyses.

Here, we systematically evaluate the prognostic significance of ETGs in sepsis. We intersected public transcriptomic datasets with curated ET gene sets, then built prediction models using an ensemble machine-learning framework that tested 108 algorithmic combinations across 12 methods. This process yielded a robust 10-gene signature. We further validated the signature in clinical samples from septic patients using qPCR, western blotting, and flow cytometry. Together, these data map a key axis of sepsis-associated immunosuppression and support ETGs as practical biomarkers for predicting 28-day mortality.

## 2 Materials and methods

### 2.1 Data acquisition and preprocessing

Gene expression datasets of septic patients, including GSE65682, GSE95233, GSE174507, GSE217700 from the Gene Expression Omnibus (GEO, https://www.ncbi.nlm.nih.gov/geo) and E-MTAB-4421, E-MTAB-4451, E-MTAB-7581 from the ArrayExpress database (https://www.ebi.ac.uk/biostudies/arrayexpress), were retrieved for analysis. Raw microarray data were processed using the Robust Multi-array Average (RMA) method for background correction and normalization. For genes represented by multiple probes, average expression values were calculated to obtain a single representative expression level.

### 2.2 Identification of endotoxin tolerance-related genes and differential expression analysis

ETGs were compiled from the GeneCards database (www.genecards.org) via keyword search “endotoxin tolerance” (22). Differentially expressed genes (DEGs) between septic patients and healthy controls were identified using the “limma” R package (23), applying a cutoff of adjusted P-value < 0.05 and |log2 fold change| > 1. Differentially expressed microRNAs (DE-miRNAs) and long non-coding RNAs (DE-lncRNAs) were similarly identified using GEO2R with the same statistical thresholds. The intersection of ETGs with DEGs defined the subset of ETGs significantly altered in sepsis. Overlaps were visualized using Venn diagrams (24).

### 2.3 Prognostic gene selection and risk score model construction

The above ETGs in sepsis were initially analyzed using univariate Cox regression with the “survival” R package as a preliminary filtering step to remove genes without prognostic signal, and prognostic features were selected based on a significance threshold of *P* < 0.01. Subsequently, 108 combinations of 12 machine learning methods, including *elastic network* (Enet), *generalised boosted regression modelling* (GBM), *gradient boosting with component-wise liner* (glmBoost), *least absolute shrinkage and selection operator* (Lasso), *linear discriminant analysis* (LDA), *naive bayes classifier* (NaiveBayes), *partial least squares regression for Cox* (plsRCox), *random forest* (RF), *ridge regression algorithm* (Ridge), *generalized linear model* (GLM), *support vector machine* (SVM), and *eXtreme Gradient Boosting* (XGBoost). The *average AUC* value of each algorithmic combination across all sets was used to evaluate the superiority of the combinations. The combination with the highest average AUC value was selected for model construction. The Pearson correlation coefficient was computed to investigate the intrinsic relationships among these ETGs, with a cutoff value of 0.2 (25). Furthermore, based on the prognostic ETGs selected via the combination with the highest *average AUC* value, multivariate Cox regression analysis was performed to construct a risk score model using the “survival” R package. The risk score of each sample was calculated using the formula: Risk Score = Σ (C_i_ * E_i_), where ***Ci*** represents the coefficient of the corresponding gene from multivariate Cox regression analysis, and ***Ei*** denotes the gene’s expression level. The prognostic effectiveness of each gene and risk score model in predicting the prognosis of septic patients was validated using Kaplan-Meier (KM) curves constructed with the “survfit” function in the “survival” R package. Patients were stratified into two groups based on the optimal cut-off value of gene expression, calculated using the “maxstat” R package.

### 2.4 Independent prognostic evaluation and nomogram development

The variables including age, and risk score from the GSE65682 dataset were analyzed together in multivariate Cox regression analysis. The “rms” R package and Cox regression were used to construct a nomogram based on these data, facilitating visualization and potential clinical application in predicting the 28-day outcomes of septic patients. The performance of the nomogram was assessed using both ROC and calibration curves.The “pROC” R package was employed for ROC curve analysis and AUC acquisition. The value of AUC represents the size of the area under the ROC curve. Typically, AUC values range from 0.5 to 1.0, with higher values indicating better performance.

### 2.5 Clinical sample collection and experimental validation

To validate the bioinformatic findings, peripheral blood was prospectively collected from 50 patients with sepsis admitted to the intensive care unit (ICU) at University-Town Hospital of Chongqing Medical University, following institutional ethical approval and written informed consent. All samples were obtained at a single early time point, within 24 h of ICU admission for sepsis according to Sepsis-3 criteria. Patients were categorized as survivors (n = 37) or non-survivors (n = 13) based on 28-day outcome. Clinical baseline characteristics of healthy controls, survivors, and non-survivors are provided in **Supplementary Table S1**.

Peripheral blood mononuclear cells (PBMCs) were isolated from 5 mL of EDTA-anticoagulated whole blood using density gradient centrifugation with Ficoll-Paque™ PLUS (GE Healthcare Life Sciences). In brief, the blood sample was diluted at a 1:1 ratio with phosphate-buffered saline (PBS), carefully layered over Ficoll-Paque, and centrifuged at 400 × g for 30 minutes at room temperature without applying the brake. Following centrifugation, the PBMC-containing layer was collected, washed twice with PBS (300 × g for 10 minutes each), and subsequently pelleted (26, 27). The resulting cell pellets were then aliquoted for downstream analyses.

#### 2.5.1 RT-qPCR

Total RNA was isolated from PBMCs using TRIzol™ Reagent (Takara Bio, Inc.) following the manufacturer’s protocol. RNA integrity and concentration were assessed by spectrophotometry (A_620_/A_280_ ratio 1.8–2.0). First-strand cDNA was synthesized from 1 µg of total RNA using the PrimeScript™ RT Reagent Kit with gDNA Eraser (RR037A; Takara Bio, Inc.) to remove genomic DNA.Quantitative PCR was conducted on a LightCycler^®^ 96 Instrument (Roche Diagnostics, Switzerland) utilizing TB Green^®^ Premix Ex Taq™ II (RR820A; Takara Bio, Inc.). Each 20 µL reaction comprised 10 µL TB Green Premix, 0.5 µL each of forward and reverse primers (sequences in **Supplementary Table 1**), 1 µL cDNA, and nuclease-free water. Thermal cycling conditions were: initial denaturation at 95°C for 30 s; 40 cycles of 95°C for 5 s and 60°C for 30 s; followed by a melt-curve step (95°C for 15 s, 60°C for 60 s, ramp to 95°C). All reactions were performed in technical triplicate. Relative mRNA levels were quantified using the 2^-^ΔΔCt method with GAPDH as the internal reference.Details of the specific primer sequences are provided in **Supplementary Table S2**.

#### 2.5.2 Western blotting

Total protein was extracted from PBMCs using RIPA lysis buffer (Beyotime, China) supplemented with protease and phosphatase inhibitors (A610425, Sangon Biotech, China). Lysates were incubated on ice for 30 min, centrifuged at 12,000 × g for 15 min at 4 °C, and supernatants collected. Protein concentration was measured by BCA assay. Equal amounts (20 µg) of protein were mixed with 5× loading buffer, denatured at 95°C for 5 min, and separated on 10% SDS–PAGE gels at 150 V for ~50 min. Proteins were then transferred onto PVDF membranes (Beyotime, China) at 100 V for 90 min. Membranes were blocked with 5% nonfat dry milk in TBST for 1.5 h at room temperature, then incubated overnight at 4°C with primary antibodies diluted in blocking buffer:FCGR1A (CD64) (1:1,000; HA601003, HUABIO, China),CX3CR1 (1:500; FNab02090, FineTest, China), TLR5 (1:1,000; ET1703-30, HUABIO, China), GAPDH (loading control; 1:1,000; GB15004, Servicebio, China), After three 10-min washes in TBST, membranes were incubated for 1 h at room temperature with HRP-conjugated secondary antibody (anti-rabbit IgG, 1:5,000; G1301, Servicebio, China) in blocking buffer. Following three additional 10-min TBST washes, signals were developed using ECL substrate and imaged on a ChemiDoc system (Bio-Rad, USA). Band intensities were quantified by densitometry in ImageJ and normalized to GAPDH. All blots were performed in triplicate.

#### 2.5.3 Flow cytometry

Freshly isolated PBMCs (1 × 10^6 cells per sample) were washed twice in PBS containing 2% fetal bovine serum (FBS; Gibco) and resuspended in 100 µL staining buffer (PBS + 2% FBS). Cells were incubated for 30 min at 4°C in the dark with the following fluorophore-conjugated monoclonal antibodies:CD14-Alexa Fluor^®^ 488 (clone HCD14; BioLegend, Cat. 341611), FCGR1A/CD64 PE (clone 10.1; BD Biosciences, Cat. 555527), CX3CR1-PE/Cy7 (clone 2A9-1; BioLegend, Cat. 341619), PD-1-APC (clone EH12.2H7; BioLegend, Cat. 367419), After staining, cells were washed twice with staining buffer (300 × g, 5 min, 4°C) and resuspended in 300 µL buffer. Data were acquired on a BD LSRFortessa™ flow cytometer (BD Biosciences) using FACSDiva™ software. Compensation was performed with single-stained compensation beads (BD CompBeads, BD Biosciences). Doublet discrimination was applied (FSC-A vs FSC-H), and dead cells were excluded using a viability dye. Fluorescence-minus-one (FMO) controls were used to define positivity thresholds. Monocytes were gated as CD14^+^ events, and the percentage and mean fluorescence intensity (MFI) of FCGR1A^+^, CX3CR1^+^, and PD-1^+^ subsets were quantified using FlowJo™ v10 (BD). Data from septic survivors and non-survivors were compared, with all samples run in duplicate.

### 2.6 Gene set enrichment analysis

The GSEA software was obtained from the online website (http://software.broadinstitute.org/gsea/index.jsp) for conducting GSEA on distinct groups The c2.cp.kegg.v7.4.symbols.gmt subcollection was downloaded from the Molecular Signatures Database (https://www.gsea-msigdb.org/gsea/downloads.jsp) for pathway and molecular mechanism evaluation (28). The minimum gene set was set at 5 and the maximum at 5000, based on gene expression profiles and phenotypic grouping. Additionally, 1000 resamples were conducted. Pathways with *P* < 0.05 and FDR < 0.25 were considered statistically significant.

### 2.7 Drug–gene interaction network and molecular docking

The prognostic ETGs were queried against the Drug–Gene Interaction Database (DGIdb) to retrieve FDA-approved and investigational compounds with reported agonist or antagonist effects (29). High-confidence drug–gene interactions were mapped and visualized in Cytoscape (v3.10.1), with nodes denoting genes or compounds and edges representing activation or inhibition relationships. For structural validation, three ETG-encoded proteins (CX3CR1, FCGR1A, and TLR5) and their top candidate ligands (e.g., valproic acid, CGP-52608) were subjected to molecular docking in AutoDock Vina (v1.1.2). Protein structures were prepared in AutoDockTools by removal of water molecules and addition of polar hydrogens (30); ligand geometries were energy-minimized using the MMFF94 force field in Open Babel. Docking simulations were performed within a 20 Å cubic grid encompassing each protein’s active or allosteric site, generating nine binding poses per complex ranked by predicted binding affinity. The highest-scoring complexes were visualized in PyMOL (v2.5.0), and key protein–ligand interactions—including hydrogen bonds and hydrophobic contacts—were annotated.

### 2.8 lncRNA-miRNA-mRNA regulatory network construction

The selected prognostic genes were considered as target genes, and we conducted a comprehensive search for miRNAs that potentially regulate these prognostic genes from the miRDB (31), TargetScan (32), and miRWalk databases (33). Only those miRNAs identified in at least one of the aforementioned databases were selected for further analysis. Additionally, we obtained information on lncRNAs that may regulate the prognostic genes or the selected miRNAs from the ENCORI database (34). Finally, utilizing Cytoscape software, we constructed and visualized a network consisting of lncRNA-miRNA-mRNA interactions.

### 2.9 Statistical analysis and data visualization

All statistical analyses and data visualizations were performed using R (v4.3.1), Cytoscape (v3.10.1), and the Sangerbox online platform. The chi-square test was used to compare 28-day mortality between high- and low-risk groups, and the Mann-Whitney U test was applied to assess differences in gene expression levels. For normally distributed continuous variables, unpaired Student’s t-tests (two groups) or one-way ANOVA (multiple groups) were used. All experiments were independently repeated three times, and data are expressed as mean ± standard deviation (SD). A two-sided P value < 0.05 was considered statistically significant.

## 3 Results

### 3.1 Identification of sepsis-associated ETGs

**Figure 1:**
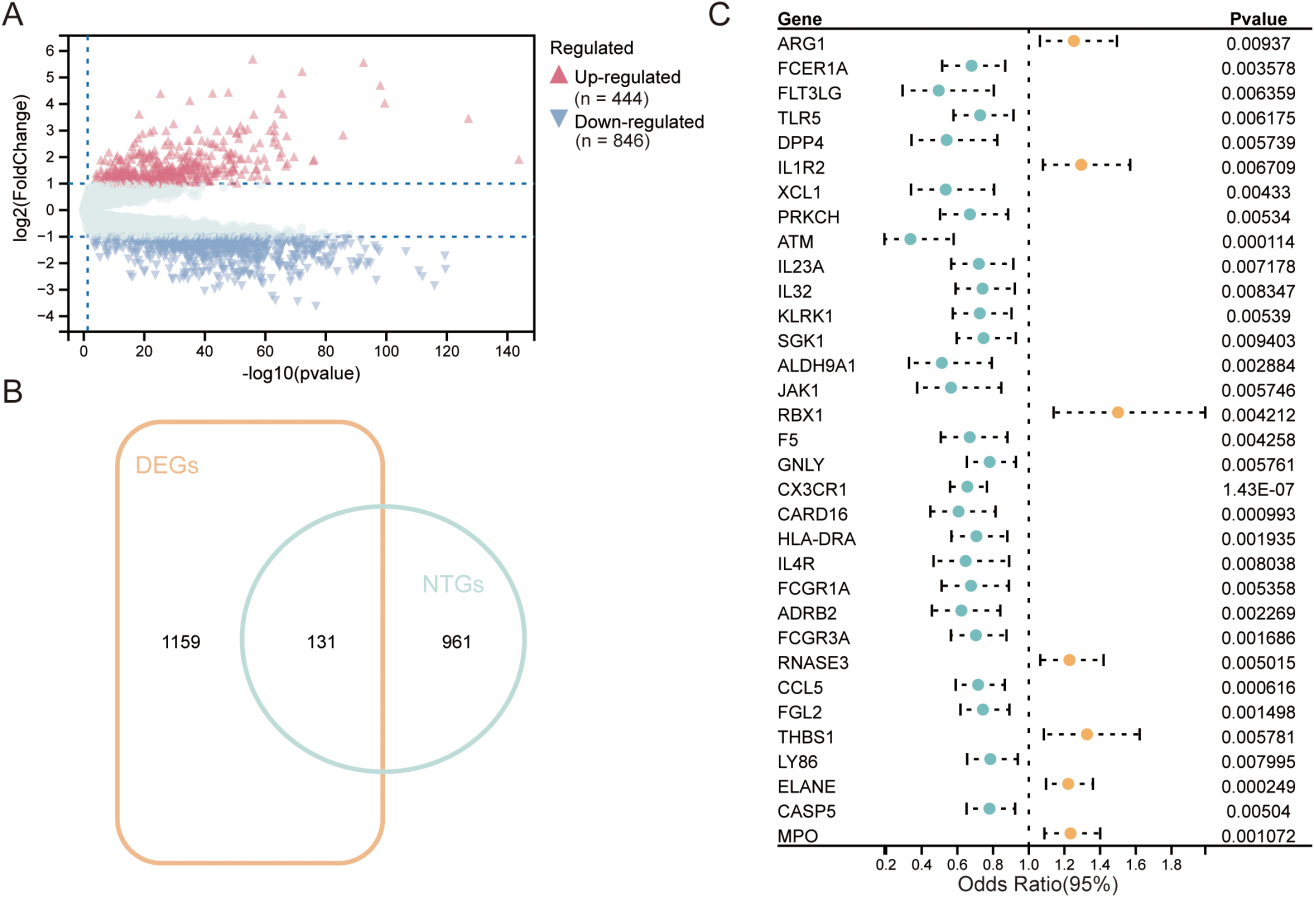
Identification of endotoxin-tolerance–related prognostic genes in sepsis. **(A)** Volcano plot of differentially expressed genes (DEGs) between patients with sepsis (n=479) and healthy controls (n=42) in GSE65682. Significance thresholds: adjusted P<0.05 and |log_2_(fold change)|>1. Points are colored by direction (upregulated, n=444; downregulated, n=846). **(B)** Venn diagram showing the overlap between DEGs and a curated endotoxin-tolerance gene set, yielding 131 ET-related genes (ETGs) taken forward. **(C)** Forest plot from univariate Cox proportional-hazards models for 28-day mortality across the 131 ETGs; 33 genes reached P<0.01. Markers indicate hazard ratio (HR) with 95% confidence interval. Blue denotes HR<1 (protective) and orange denotes HR>1 (risk).

Using the GSE65682 dataset comprising 479 septic patients and 42 healthy controls, we identified 1,310 differentially expressed genes (DEGs) (**Figure 1A**). Cross-referencing these DEGs with a curated list of endotoxin tolerance-related genes (ETGs) yielded 131 sepsis-associated ETGs (**Figure 1B**). Univariate Cox regression analysis further pinpointed 33 ETGs significantly correlated with 28-day survival in septic patients (**Figure 1C**), highlighting their prognostic potential in sepsis progression and outcome prediction.

### 3.2 Construction of a 28-day prognostic risk score model based on ETGs

**Figure 2:**
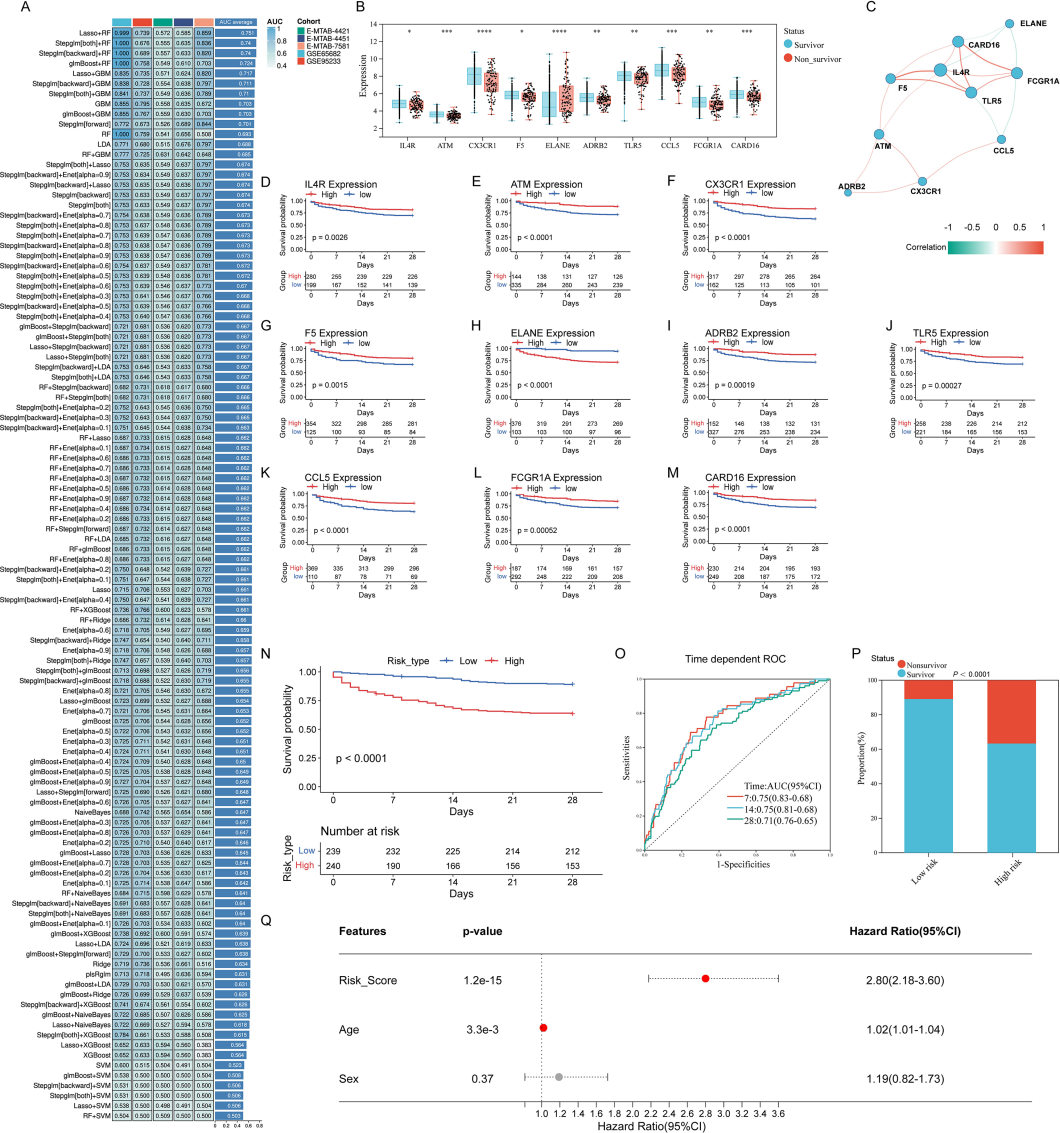
Construction and performance of a 10-gene ETG-based risk score for 28-day mortality in sepsis. **(A)** Model leaderboard across 108 algorithmic combinations (12 methods). Training: GSE65682; internal test: GSE95233; external validations: E-MTAB-7581/4451/4421. The Lasso+Random Forest (Lasso+RF) model achieved the highest mean AUC = 0.751 across cohorts and was selected for downstream analyses. **(B)** Differential expression of the 10 model genes (IL4R, ATM, CX3CR1, F5, ELANE, ADRB2, TLR5, CCL5, FCGR1A, CARD16) in survivors vs. non-survivors (two-sided Wilcoxon test; ****P<0.0001, ***P<0.001, **P<0.01, *P<0.05). **(C)** Pearson correlation network among the 10 genes; edge color indicates sign (cyan, negative; salmon, positive) and width indicates magnitude. **(D–M)** Gene-wise Kaplan–Meier curves (patients split by median expression); log-rank P values shown. **(N)** Kaplan–Meier survival stratified by the ETG risk score (derived from a multivariable Cox model using the 10 genes; coefficients in Methods). Patients were dichotomized by the cohort mean (−9.1886) into low-risk (n=239) and high-risk (n=240) groups (log-rank P<0.0001). Numbers at risk are displayed. **(O)** Time-dependent ROC curves for the risk score at 7/14/28 days with AUCs 0.75 (95% CI 0.68–0.83), 0.75 (0.68–0.81), and 0.71 (0.65–0.76), respectively. **(P)** Outcome distribution by risk group (survivor vs. non-survivor; χ² test P<0.0001). **(Q)** Multivariable Cox analysis including risk score, age, and sex. The risk score remained an independent predictor (HR 2.80, 95% CI 2.18–3.60; P = 1.2×10^−15); age showed a modest effect (HR 1.02, 95% CI 1.01–1.04); sex was not significant (HR 1.19, 95% CI 0.82–1.73).

Using the expression profiles of 33 features identified by univariate Cox regression, we constructed prognostic models to predict 28-day survival in septic patients. A total of 108 algorithmic combinations derived from 12 machine learning methods were applied to the GSE65682 dataset as the training set, with GSE95233 as the test set and E-MTAB-7581, E-MTAB-4451, and E-MTAB-4421 as independent validation cohorts. The Lasso combined with Random Forest (Lasso+RF) model achieved the highest average AUC of 0.751 across these datasets and was selected for subsequent analyses (**Figure 2A**).

From this model, 10 prognostic genes—*IL4R, ATM, CX3CR1, F5, ELANE, ADRB2, TLR5, CCL5, FCGR1A, and CARD16*—were identified, showing significant differential expression between survivors and non-survivors (**Figure 2B**). Pearson correlation analysis revealed complex interactions among these genes; for instance, FCGR1A correlated positively with CARD16, IL4R, and TLR5, but negatively with ELANE and CCL5, suggesting intricate regulatory networks involved in endotoxin tolerance during sepsis progression (**Figure 2C**).The interrelationship among these 10 genes appeared to be complex. For instance, *FCGR1A* showed positive correlations with *CARD16*, *IL4R*, and *TLR5* but negative correlations with *ELANE* and *CCL5*, suggesting intricate regulatory mechanisms involved in ET during sepsis development.

Based on the expression profiles of these 10 genes, we conducted KM curves to illustrate their prognostic characteristics in sepsis development. Importantly, all 10 genes exhibited significant prognostic roles (**Figures 2D–M**). Furthermore, a risk score model was constructed using multivariate Cox regression analysis. The risk score for each septic patient was calculated using the following formula: risk score = (0.032893669062406 * Expression*_IL4R_* -0.309137796227894 * Expression*_ATM_* - 0.237720552080183 * Expression*_CX3CR1_* - 0.0793200673882997 * Expression*_F5_* + 0.0968903151266514 * Expression*_ELANE_* - 0.224354506424999 * Expression*_ADRB2_* - 0.181943063486774 * Expression*_TLR5_* - 0.252376566454744 * Expression*_CCL5_* - 0.17720306936071 * Expression*_FCGR1A_* - 0.123979346077383 * Expression*_CARD16_*). Based on the mean value of the risk score (-9.188638104), patients in the training cohort were stratified into a low-risk group (n = 239) and a high-risk group (n = 240). The KM curve showed a significantly higher survival rate in the low-risk group than in the high-risk group (**Figure 2N**). Time-dependent ROC analysis of the risk score showed an AUC of 0.75 (95% CI: 0.68 to 0.83), 0.75 (95% CI: 0.68 to 0.81), and 0.71 (95% CI: 0.65 to 0.76) for survival at intervals of 7, 14, and 28 days, respectively (**Figure 2O**). Additionally, death events were more likely to occur in the high-risk group among septic patients (**Figure 2P**). The risk score was identified as an independent prognostic predictor for septic patients through multivariate Cox regression analysis (**Figure 2Q**).

Collectively, these results demonstrate that the 10-gene ETG-based risk model robustly stratifies septic patients by mortality risk, providing a valuable tool for early prognostic assessment.

### 3.3 Construction of a nomogram based on the risk score

**Figure 3:**
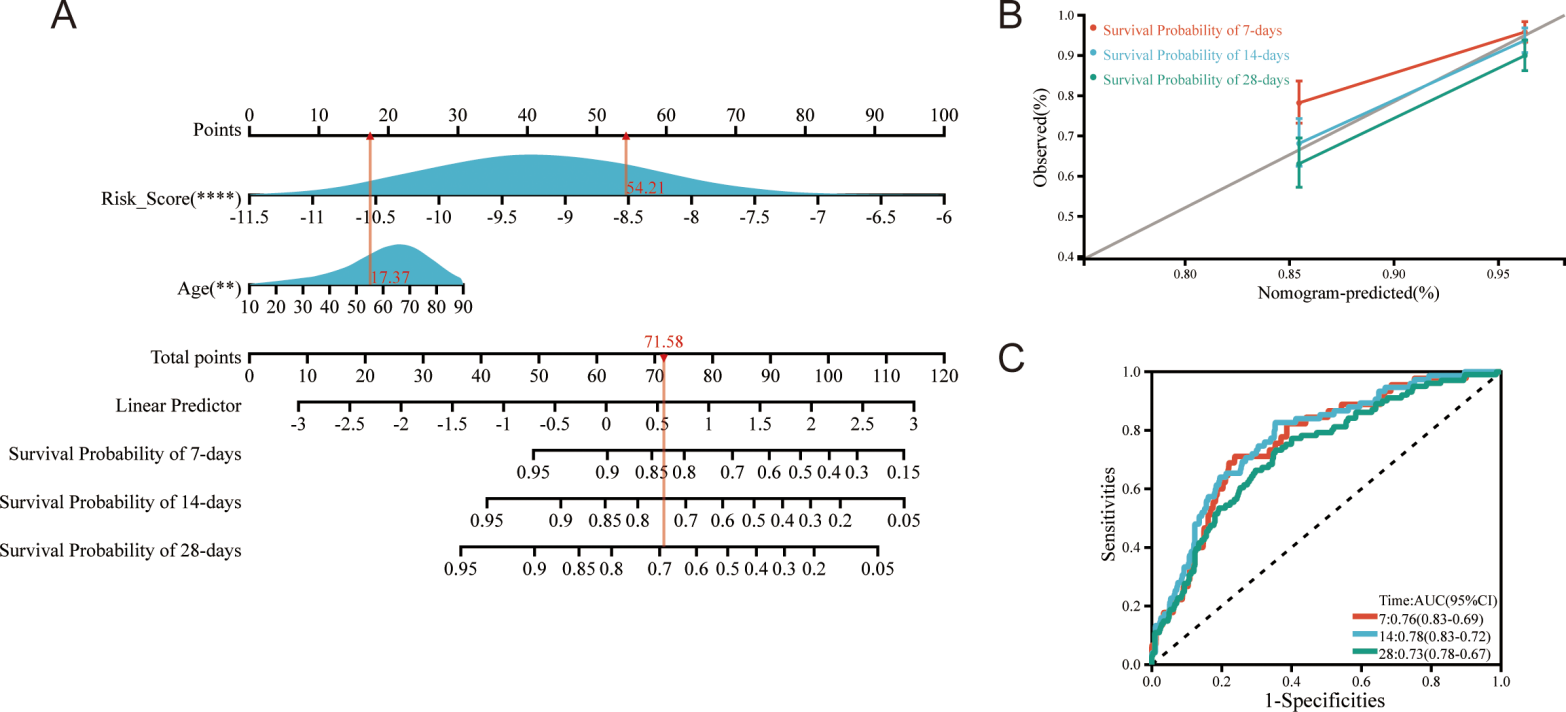
Nomogram integrating the ETG-based risk score with age for short-term survival prediction in sepsis. **(A)** Two-variable nomogram assigning points to age and the ETG risk score; the sum (Total points) maps to predicted probabilities of survival at 7, 14, and 28 days. **(B)** Calibration plots for 7-, 14-, and 28-day survival showing agreement between predicted and observed outcomes after 1,000-bootstrap internal validation (ideal 45° line shown). **(C)** Time-dependent ROC curves for the nomogram at 7/14/28 days with AUCs 0.76 (95% CI 0.69–0.83), 0.78 (0.72–0.83), and 0.73 (0.67–0.78), respectively.

To enhance clinical applicability and prognostic accuracy, we developed a two-variable nomogram that combines age with the ETG-based risk score (**Figure 3A**). Internal bootstrap validation (1,000 resamples) showed close agreement between predicted and observed survival at 7, 14, and 28 days (**Figure 3B**). Discrimination was acceptable to good: time-dependent ROC analyses yielded AUCs of 0.76 (95% CI 0.69–0.83), 0.78 (0.72–0.83), and 0.73 (0.67–0.78) at 7, 14, and 28 days, respectively (**Figure 3C**). Harrell’s C-index was 0.782, indicating consistent concordance between predicted risk and observed outcomes. Overall, the nomogram provides a simple and reproducible tool for short-term risk stratification in sepsis.

### 3.4 Functional enrichment analysis of risk stratification and prognostic ETGs

**Figure 4:**
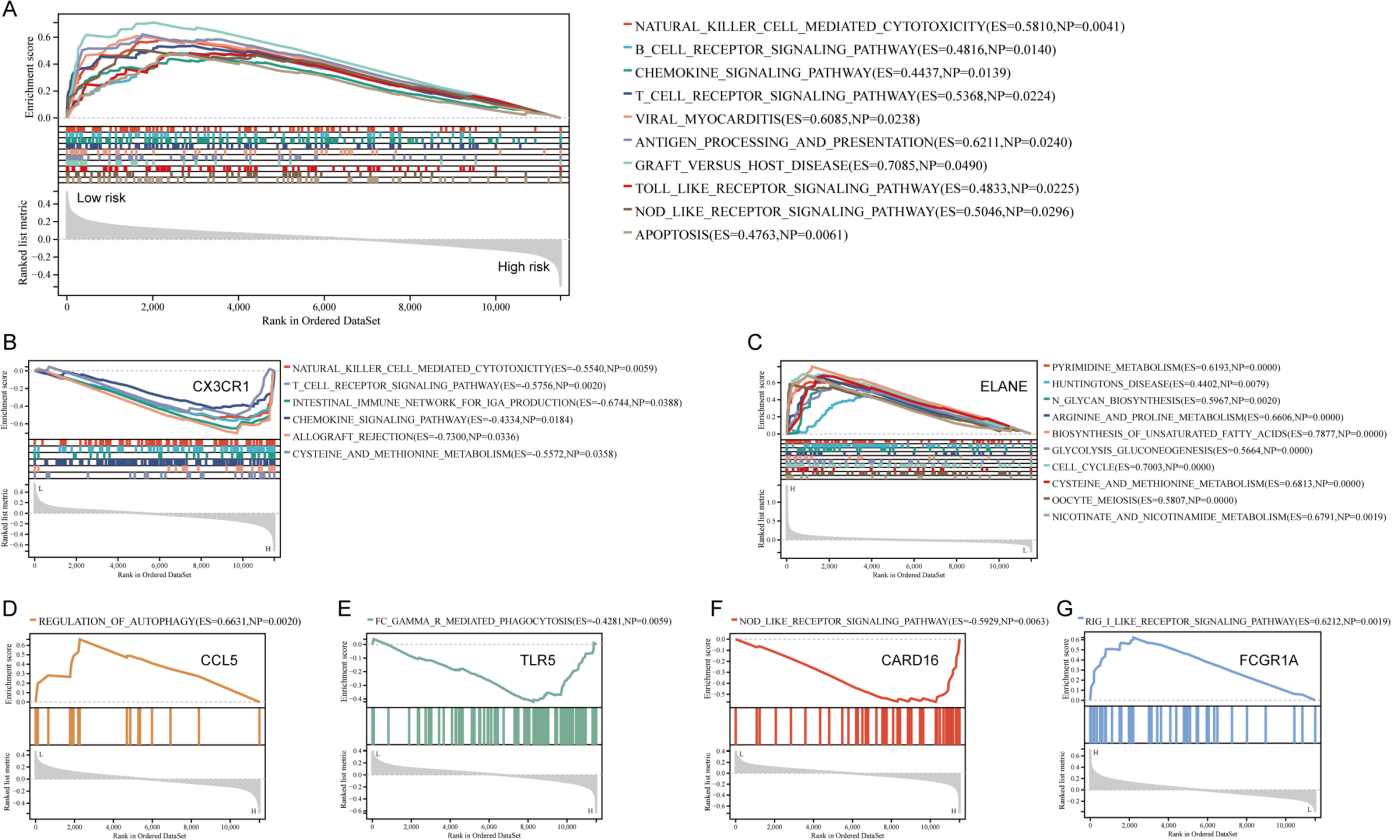
Functional enrichment of risk strata and individual prognostic ETGs. **(A)** KEGG gene set enrichment analysis (GSEA) comparing low- versus high-risk groups defined by the ETG risk score. Low-risk patients showed enrichment of immune/inflammatory pathways, including natural killer cell–mediated cytotoxicity, B-cell receptor signaling, chemokine signaling, T-cell receptor signaling, antigen processing and presentation, and Toll-like receptor signaling. Enrichment is summarized by normalized enrichment score (NES) and multiple-testing–adjusted *q* values. **(B–G)** KEGG pathway associations for selected prognostic ETGs: **(B)** CX3CR1 (natural killer cell–mediated cytotoxicity, T-cell receptor signaling, intestinal immune network for IgA production, chemokine signaling); **(C)** ELANE (pyrimidine metabolism, N-glycan biosynthesis, arginine and proline metabolism, biosynthesis of unsaturated fatty acids); **(D)** CCL5 (autophagy); **(E)** TLR5 (Fc-gamma R–mediated phagocytosis); **(F)** CARD16 (NOD-like receptor signaling); **(G)** FCGR1A (RIG-I–like receptor signaling). Dot/bubble size reflects gene ratio; color encodes adjusted *P*.

To further investigate the biological differences between low-risk and high-risk groups, we conducted KEGG pathway analysis using GSEA. The results revealed that the low-risk group exhibited enrichment in multiple immune and inflammation-related pathways, including natural killer cell mediated cytotoxicity, B cell receptor signaling pathway, chemokine signaling pathway, T cell receptor signaling pathway, antigen processing and presentation, as well as Toll-like receptor signaling pathway (**Figure 4A**). Additionally, we investigated potential biological pathways associated with the prognostic genes. In terms of KEGG analysis, *CX3CR1* primarily participates in natural killer cell mediated cytotoxicity, T cell receptor signaling pathway, intestinal immune network for IgA production, and chemokine signaling pathway (**Figure 4B**). *ELANE* is mainly involved in pyrimidine metabolism, N glycan biosynthesis, arginine and proline metabolism, and biosynthesis of unsaturated fatty acids (**Figure 4C**). *CCL5* primarily regulates autophagy (**Figure 4D**). *TLR5* plays a significant role in FC gamma R-mediated phagocytosis (**Figure 4E**). *CARD16* is predominantly involved in NOD-like receptor signaling pathway (**Figure 4F**). *FCGRIA* mainly participates in Rig-I like receptor signaling pathway (**Figure 4G**). While the results about *ATM*, *IL4R*, *F5*, and *ADRB2* were not statistically significant in KEGG pathway analysis using GSEA.

(Not shown) ATM, IL4R, F5, and ADRB2 did not reach significance under the prespecified multiple-testing threshold.

### 3.5 Experimental validation in clinical sepsis cohort

To verify translational relevance, peripheral blood was obtained from 50 ICU patients with sepsis (37 survivors, 13 non-survivors). PBMCs were isolated for mRNA/protein assays, and CD14^+^ monocytes were profiled by flow cytometry.

**Figure 5:**
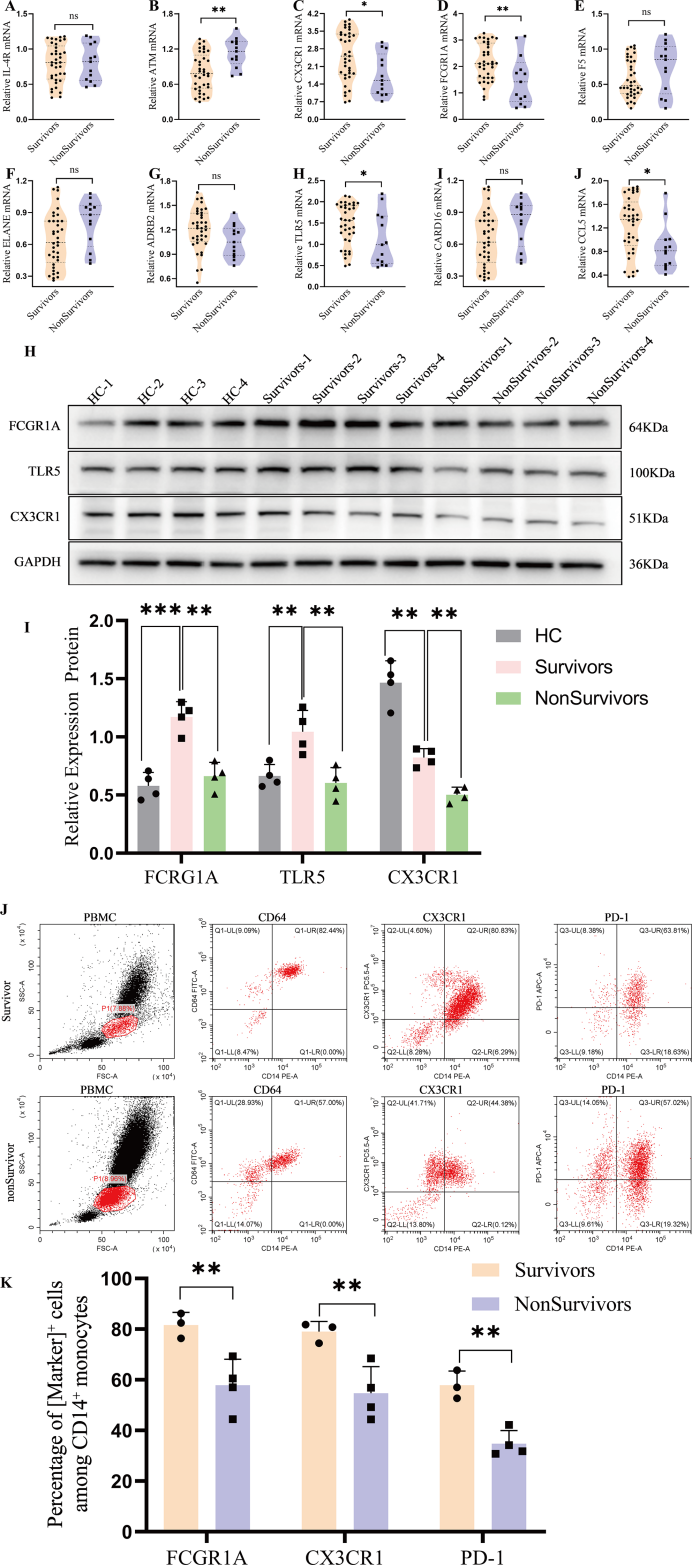
Experimental validation of endotoxin-tolerance–related markers in a clinical sepsis cohort. **(A–G)** RT-qPCR in peripheral blood mononuclear cells (PBMCs) from ICU patients with sepsis (n=50; survivors n=37, non-survivors n=13). FCGR1A, TLR5, and CX3CR1 mRNA levels were significantly lower in non-survivors than survivors, consistent with discovery-set predictions (panels as labeled). **(H–I)** Immunoblot analysis of PBMC lysates from representative survivors and non-survivors confirming reduced FCGR1A, TLR5, and CX3CR1 protein abundance in non-survivors; healthy controls (HCs) are shown as an external reference and were not included in statistical comparisons. **(J–K)** Flow cytometry of CD14^+^monocytes showing lower proportions of FCGR1A^+^, CX3CR1^+^, and PD-1^+^cells in non-survivors versus survivors (P<0.01). Representative gating and group summaries are shown. ***p < 0.001, **p < 0.01, *p <0.05; ns, not significant.

RT-qPCR analysis revealed significantly lower mRNA expression of FCGR1A, TLR5, and CX3CR1 in non-survivors compared with survivors (**Figures 5A–G**), consistent with the transcriptomic predictions. To confirm protein-level differences, immunoblotting of PBMC lysates from representative subgroups likewise demonstrated reduced protein expressiocn of these three targets in non-survivors relative to survivors, with healthy controls included as a reference (**Figures 5H, I**), consistent with attenuated innate-immune signaling in poor-outcome patients.

At the single-cell level, flow cytometry of CD14^+ monocytes showed lower proportions of FCGR1A^+, CX3CR1^+, and PD-1^+ cells in non-survivors versus survivors (**Figures 5J, K**; P < 0.01), indicating impaired immune activation and antigen-presenting capacity.

To further assess their immune relevance at the single-cell level, flow cytometry analysis was performed on CD14^+^ monocytes. We quantified the percentage of CD14^+^ monocytes expressing FCGR1A, CX3CR1, and PD-1. Results showed that the proportions of FCGR1A^+^, CX3CR1^+^, and PD-1^+^ monocytes were significantly reduced in non-survivors compared with survivors (p < 0.01; **Figures 5J, K**), suggesting impaired immune activation and antigen-presenting capacity. These results reinforce the functional relevance of endotoxin tolerance in determining sepsis prognosis and provide cellular-level evidence supporting the bioinformatic findings.

Taken together, these clinical validations indicate that the ET-related gene signature has prognostic utility and captures underlying immunological dysfunction in sepsis.

### 3.6 Construction of lncRNA–miRNA–mRNA regulatory network

**Figure 6:**
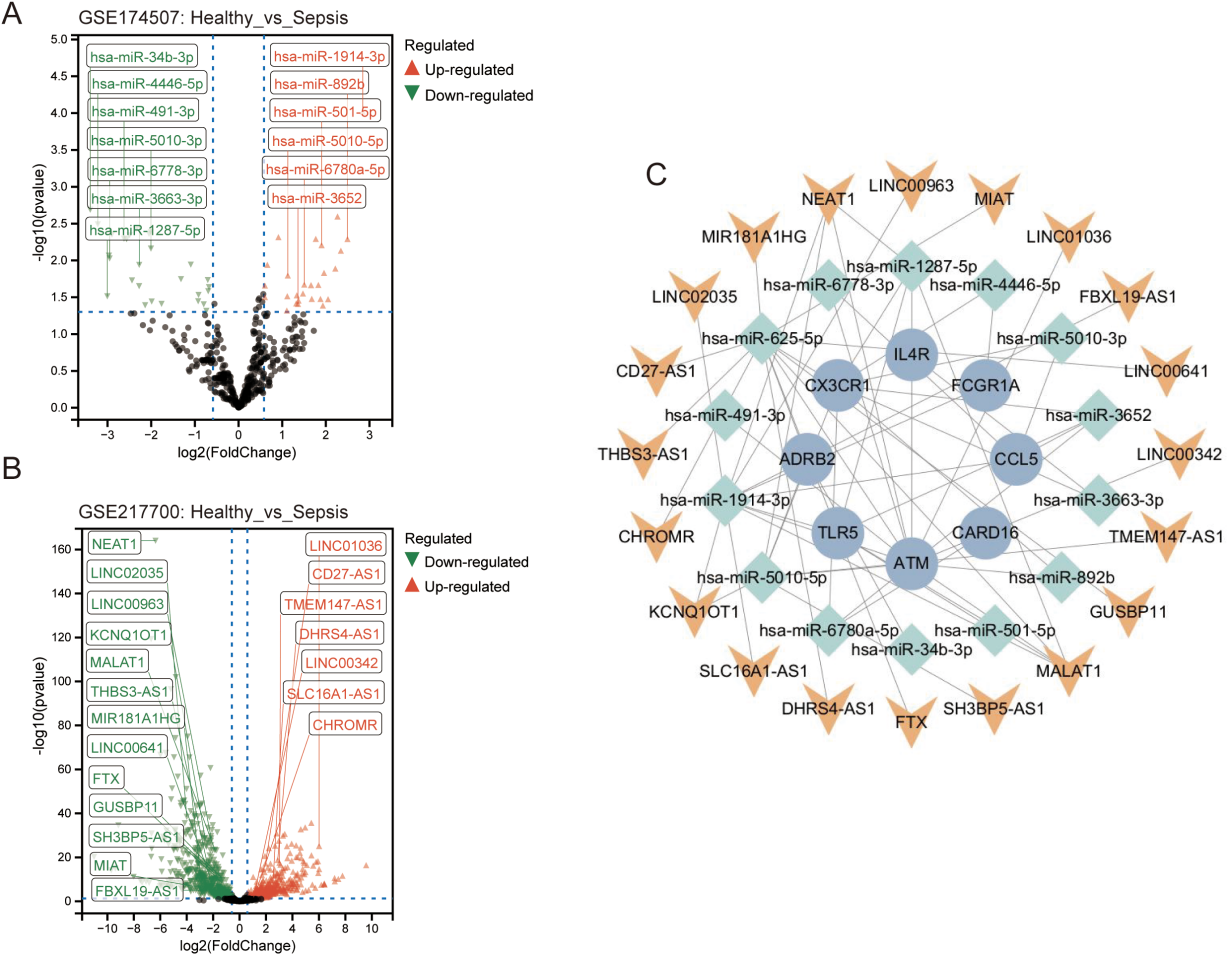
ceRNA network linking non-coding RNAs to prognostic ETGs in sepsis. **(A)** Differentially expressed miRNAs in whole blood (GSE174507; thresholds per Methods). **(B)** Differentially expressed lncRNAs (GSE217700). **(C)** Integrated lncRNA–miRNA–mRNA (ceRNA) network connecting DE-lncRNAs/DE-miRNAs with the 10 prognostic ETGs based on miRDB/TargetScan/miRWalk and ENCORI. Representative axes include NEAT1/miR-1287-5p/CX3CR1, MIR181A1HG/miR-491-3p/ADRB2, and LINC00342/miR-3663-3p/CARD16.

To investigate the post-transcriptional regulatory mechanisms of the identified prognostic genes, a comprehensive competing endogenous RNA (ceRNA) network was constructed. Initially, differential expression analyses were conducted to identify sepsis-associated miRNAs and lncRNAs using publicly available datasets. Specifically, 14 differentially expressed miRNAs (DE-miRNAs) were obtained from GSE174507 (**Figure 6A**), comprising both upregulated (e.g., hsa-miR-1914-3p, hsa-miR-892b) and downregulated (e.g., hsa-miR-4446-5p, hsa-miR-491-3p) candidates. Concurrently, 20 differentially expressed lncRNAs (DE-lncRNAs) were identified in GSE217700, including NEAT1, LINC02035, and MALAT1, with the majority being downregulated in patients with sepsis (**Figure 6B**).

Subsequently, the miRDB, TargetScan, and miRWalk databases were utilized to predict potential miRNAs targeting the 10 prognostic immune-related genes (ETGs). These predicted miRNAs were then intersected with the DE-miRNAs to ensure disease relevance. The ENCORI database was further employed to identify upstream lncRNAs that interact with these miRNAs and/or ETGs.

All validated lncRNA–miRNA and miRNA–mRNA interactions were integrated into a lncRNA–miRNA–mRNA tripartite regulatory network (**Figure 6C**), revealing several prominent ceRNA axes, such as:NEAT1/hsa-miR-1287-5p/CX3CR1, MIR181A1HG/hsa-miR-491-3p/ADRB2,LINC00342/hsa-miR-3663-3p/CARD16.

These interactions suggest that multiple lncRNAs may regulate endotoxin tolerance and immune responses in sepsis by competitively binding to regulatory miRNAs, thereby indirectly modulating key mRNA expression levels. Collectively, these findings offer mechanistic insights into how non-coding RNAs may fine-tune the expression of critical immune genes in sepsis, potentially identifying novel therapeutic targets or biomarkers for clinical applications.

### 3.7 Molecular docking supports therapeutic potential of key ETGs

**Figure 7:**
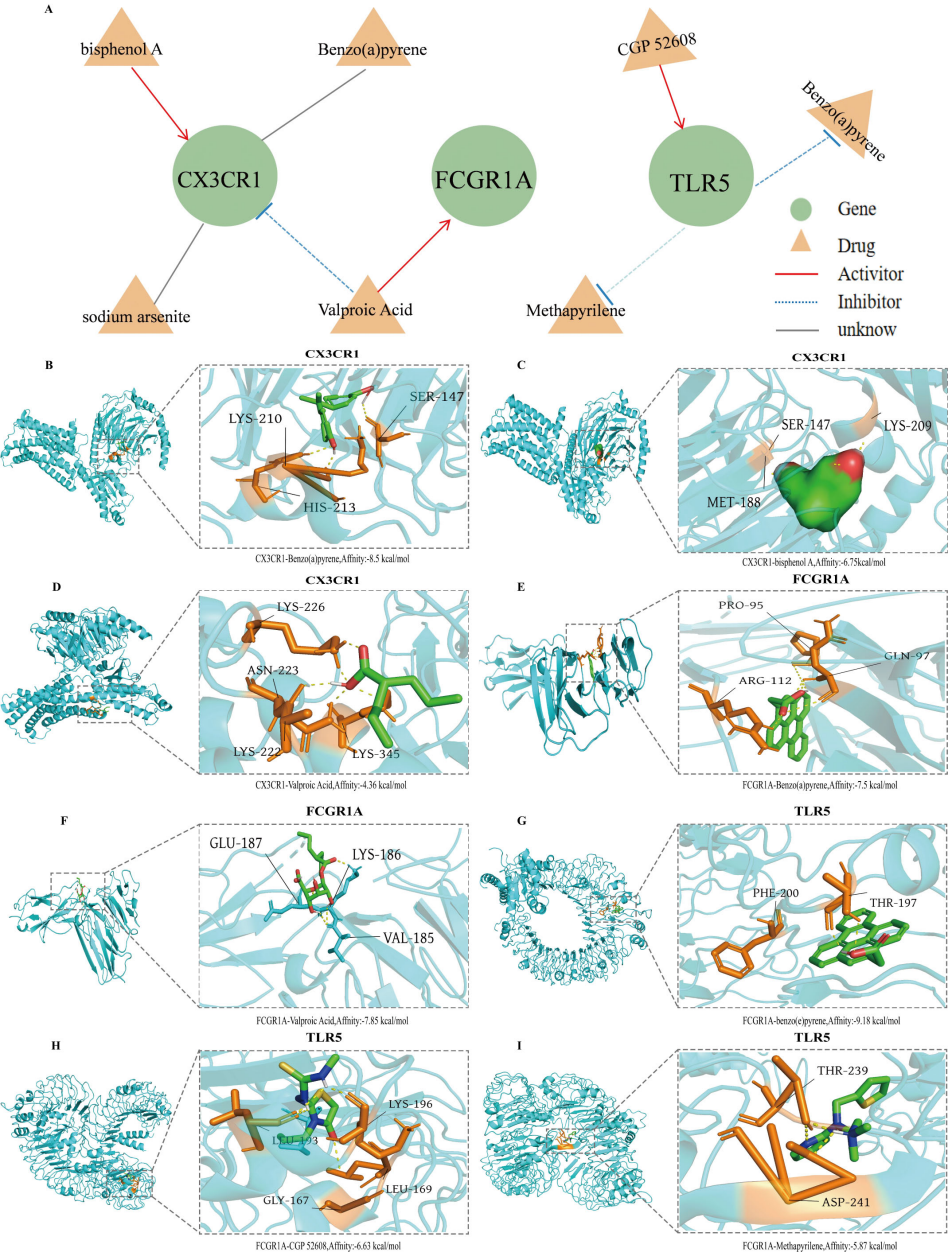
Drug–gene interactions and structure-based docking for key ETGs. **(A)** DGIdb-derived drug–gene network for CX3CR1, FCGR1A (CD64), and TLR5. Triangles denote compounds; edges indicate reported direction (red, activator; blue dashed, inhibitor; grey, unknown). **(B–D)** Docking to CX3CR1: bisphenol A (−8.5 kcal/mol) forming H-bonds with Ser147/His213/Lys210 **(B)**; benzo[a]pyrene (−7.6 kcal/mol) occupying a pocket near Ser147/Lys209/Met188 **(C)**; valproic acid (−6.3 kcal/mol) contacting Asn223/Lys222/Lys345 **(D)**. **(E, F)** Docking to FCGR1A: benzo[a]pyrene (−7.1 kcal/mol) within a hydrophobic cavity formed by Pro95/Arg112/Gln97 **(E)**; valproic acid (−7.5 kcal/mol) interacting with Val185/Lys186/Glu187 **(F)**. **(G–I)** Docking to TLR5: benzo[a]pyrene (−6.1 kcal/mol) near Phe200/Thr197 **(G)**; CGP-52608 (−6.5 kcal/mol) contacting Lys196/Gly167/Leu169 **(H)**; methapyrilene (−5.8 kcal/mol) forming H-bonds with Thr239/Asp241 **(I)**.

To explore pharmacological relevance for the clinically validated ETGs, we queried DGIdb for small-molecule interactions and constructed a drug–gene network (**Figure 7A**). The network includes compounds annotated as activators (e.g., valproic acid, CGP-52608, methapyrilene) or inhibitors (e.g., sodium arsenite), while others (e.g., benzo[a]pyrene, bisphenol A) have context-dependent or uncertain directionality; each target was linked to multiple candidate ligands, suggesting tractable binding sites rather than establishing efficacy.

We then performed structure-based docking. For CX3CR1, bisphenol A showed the most favorable predicted affinity (−8.5 kcal/mol) with hydrogen bonds to Ser147, His213, and Lys210 (**Figure 7B**); benzo[a]pyrene (−7.6 kcal/mol) and valproic acid (−6.3 kcal/mol) occupied pockets involving Met188, Asn223, and Lys345 (**Figures 7C, D**). FCGR1A displayed favorable binding to benzo[a]pyrene (−7.1 kcal/mol) within a hydrophobic pocket (Pro95/Arg112/Gln97; **Figure 7E**) and to valproic acid (−7.5 kcal/mol) via contacts with Val185, Lys186, and Glu187 (**Figure 7F**). For TLR5, benzo[a]pyrene docked with moderate affinity (−6.1 kcal/mol) near Phe200/Thr197 (**Figure 7G**), whereas CGP-52608 (−6.5 kcal/mol) and methapyrilene (−5.8 kcal/mol) formed contacts around Lys196/Gly167/Leu169 and Thr239/Asp241, respectively (**Figures 7H, I**).

Taken together, the docking analyses support the structural feasibility of several ETG–ligand pairs and nominate CX3CR1, FCGR1A, and TLR5 as ligandable nodes within the ET axis, with candidate chemotypes such as valproic acid and CGP-52608. Because docking scores indicate putative binding rather than functional modulation, orthogonal biophysical assays (SPR/ITC/CETSA) and cell-based target-engagement/phenotypic tests, followed by *in vivo* evaluation, are required before any therapeutic inference.

## 4 Discussion

Despite considerable advances in identifying biomarkers such as inflammatory factors and miRNAs, prognostic prediction of sepsis remains challenging due to its complex etiology and pathophysiology (35, 36). To address this, we assessed the prognostic value of endotoxin tolerance-related genes (ETGs) through univariate Cox regression and extensive machine learning model screening, identifying a robust 10-gene signature including IL4R, ATM, CX3CR1, and FCGR1A that accurately predicts 28-day mortality. Based on this signature, we constructed a prognostic risk score stratifying patients into high- and low-risk groups, with distinct survival outcomes. Functional enrichment revealed immune response pathways enriched in low-risk patients. Furthermore, we developed a nomogram demonstrating excellent predictive efficacy and constructed drug-gene and lncRNA-miRNA-mRNA networks providing novel therapeutic insights. This comprehensive, multi-dimensional approach establishes a biologically grounded framework linking immune dysregulation with clinical outcomes, offering promising avenues for personalized risk stratification and targeted interventions in sepsis.

Building upon the established prognostic utility of the ETG-based model, our multi-layered validation across transcriptomic, proteomic, and single-cell assays revealed marked immune differences between risk strata. In CD14^+ monocytes from high-risk non-survivors, CX3CR1 and FCGR1A (CD64) were significantly reduced—a biologically coherent pattern: loss of CX3CR1 is consistent with impaired patrolling/trafficking of nonclassical monocytes that underpins intravascular immune surveillance, whereas lower FCGR1A indicates diminished FcγRI-mediated immune-complex capture with downstream routing toward antigen processing and MHC-II presentation pathways (37–41). Taken together, these features align with an endotoxin-tolerance–like immunosuppressive state in circulating monocytes. Endotoxin tolerance itself represents a coordinated, multi-layer reprogramming of innate immunity rather than a single linear pathway, and the genes within our signature capture distinct facets of this tolerant phenotype. FCGR1A and CX3CR1 reflect monocyte activation states known to decline during ET-associated hyporesponsiveness, consistent with our flow-cytometric findings in non-survivors. Notably, PD-1 was assessed on CD14^+^ monocytes rather than on T cells, and non-survivors displayed concomitantly reduced proportions of FCGR1A^+^, CX3CR1^+^, and PD-1^+^ monocytes, suggesting a global loss or deactivation of functionally competent monocyte subsets rather than selective PD-1 up-regulation. IL1R2, as a decoy receptor, participates in negative feedback that attenuates IL-1–driven inflammation, whereas suppression of TLR5 aligns with broader desensitization of pattern-recognition signaling in tolerant monocytes. Other components—including MAL, TNFAIP6, IER3, and NR4A1—have been implicated in stress adaptation, feedback inhibition, or metabolic restraint during repeated inflammatory stimulation. Together, these genes represent complementary modules of the ET response, supporting the concept that the prognostic value of the ETG-based model derives from its ability to capture multiple dimensions of monocyte immune hyporesponsiveness. This mechanistic interpretation reinforces the view that immunosuppression—rather than early hyperinflammation—is a major determinant of sepsis mortality (42–44). The broader exhaustion phenotype—including T-cell dysfunction and reduced monocyte HLA-DR reported in prior studies—is concordant with this interpretation and motivates therapeutic strategies aimed at restoring host immune competence (41, 45, 46).

Our study advances sepsis prognostication beyond prior transcriptomic and clinical scoring models by focusing explicitly on ETGs, which provide clearer mechanistic insight and translational relevance. Existing models such as the Sepsis Response Signature (SRS) and MARS endotypes classify patients primarily based on broad transcriptomic alterations without specific reference to immune tolerance pathways (47–49). In contrast, our ETG-centric approach identifies discrete molecular targets intimately involved in monocyte and macrophage dysfunction—the principal mediators of immune tolerance in sepsis (42, 44). The rigorous application of LASSO combined with Random Forest machine learning algorithms enabled robust feature selection and model generalizability across multiple independent cohorts, effectively mitigating risks of overfitting and enhancing reproducibility, a common limitation of earlier models (50–52). Importantly, our integration of biological validation in a prospective ICU cohort by RT-qPCR, Western blotting, and flow cytometry bridges the gap between computational prediction and biological reality, bolstering the translational robustness of the ETG signature.Functional analysis and experimental validation of individual ETGs substantially reinforce the biological plausibility of our prognostic signature. CX3CR1, a chemokine receptor central to monocyte trafficking and immune surveillance, has been implicated in host defense mechanisms against infections; its reduced expression in non-survivors reflects impaired monocyte migration and heightened vulnerability to secondary infections (39, 53–55). Similarly, FCGR1A (CD64), a high-affinity Fc gamma receptor pivotal for IgG-mediated phagocytosis, serves as a critical effector of innate immunity. The observed downregulation aligns with prior studies associating decreased CD64 levels with worsened clinical outcomes in severe infections (40, 56–59). Additionally, TLR5, a pattern recognition receptor recognizing bacterial flagellin, was suppressed in high-risk patients, likely compromising pathogen detection and facilitating persistent infection, thereby exacerbating sepsis severity (60, 61). These gene-level insights illuminate critical nodes of immune dysfunction driving endotoxin tolerance and highlight tangible targets for future therapeutic development.

Beyond biomarker discovery, the integration of the ETG-derived risk score with key clinical variables such as age into a nomogram substantially enhances its translational utility (50, 62). Demonstrating strong predictive performance (C-index = 0.782) and favorable decision curve analysis outcomes, our model surpasses conventional clinical scoring systems by embedding mechanistic insights directly tied to immune status (49, 51, 52). This precision enables early stratification of septic patients, facilitating timely and tailored immunomodulatory interventions for individuals at highest risk. Concordant validation across transcriptomic, proteomic, and single-cell resolution data further reinforces the clinical reliability and applicability of this prognostic framework, bridging computational insights to actionable clinical tools (42).

Extending the mechanistic understanding, our construction of a comprehensive competing endogenous RNA (ceRNA) network reveals epigenetic layers modulating ETG expression. Notably, the NEAT1/miR-1287-5p/CX3CR1 axis—predicted by our network—exemplifies how long non-coding RNAs (lncRNAs) can competitively modulate microRNA activity and thereby influence key immune genes (63–65). This is consistent with contemporary views that lncRNAs act as master regulators of inflammatory and immunosuppressive states during bacterial infection and immune activation (66). Collectively, these regulatory circuits introduce therapeutic avenues beyond protein-coding targets and underscore the complexity of immune reprogramming in sepsis.

Complementing these regulatory insights, molecular docking analyses—grounded in curated drug–gene resources and standard docking workflows—nominated valproic acid (FDA-approved) and CGP-52608 (a RORα ligand) as candidates showing favorable predicted binding to ETG-encoded targets (FCGR1A, CX3CR1, TLR5) (29, 67). Structural models used for docking were supported by high-resolution complexes (CX3CR1–Gi, TLR5–flagellin, and FcγRI–Fc), ensuring physically plausible binding poses (68–70). These in silico results, together with literature on valproic acid immune-modulatory actions in shock/sepsis models and the immunologic role/ligandability of RORα (for CGP-52608), suggest a rationale for drug repurposing to mitigate endotoxin-tolerance–associated immunosuppression [7–9]. Nevertheless, docking scores indicate putative binding rather than functional modulation; orthogonal target-engagement and biophysical verification (e.g., CETSA, SPR/ITC), followed by *in vitro* and *in vivo* studies, are required prior to any therapeutic inference.

Despite these strengths, several limitations warrant consideration. Although our model was rigorously validated in multiple independent transcriptomic cohorts and a prospective clinical sample, the clinical validation sample size (n=50) remains modest, limiting broad generalizability. best practice and reporting guidance emphasize larger, well-powered external validations (50, 71). Larger, multicenter prospective studies are essential for external validation and refinement of prognostic accuracy. In addition, all discovery-stage transcriptomic datasets were generated from bulk whole-blood samples, so the ETG–DEG overlap reflects composite leukocyte expression and cannot resolve the precise cellular origin of ET-related signals. Moreover, the reliance on retrospective public datasets constrains availability of detailed clinical metadata such as infection source, pathogen type, and comorbidities, which may confound results. Future studies incorporating more granular clinical data will enhance model precision. Furthermore, while molecular docking offers valuable structural insights, functional studies are needed to verify the efficacy and safety of proposed drug candidates, ideally employing cellular and animal models. Lastly, endotoxin tolerance is a dynamic immunological state; serial monitoring of immune profiles over time would elucidate temporal changes in ETG expression and immune status, refining prognostic power and informing therapeutic timing.

In summary, our comprehensive multi-omics and machine learning approach elucidates the central role of endotoxin tolerance-related immunosuppression in sepsis prognosis, culminating in a robust, clinically validated 10-gene risk model. By integrating transcriptomic biomarkers, immune regulatory networks, and therapeutic predictions, this study establishes a novel paradigm for personalized sepsis management. Future research should focus on large-scale prospective validation, mechanistic dissection of ETG regulation, and clinical trials evaluating reversal of immune tolerance as a promising therapeutic strategy to improve sepsis outcomes.

## 5 Conclusion

In summary, we identified the predictive value of ETGs and developed a prognostic predictive risk score for 28-day mortality among septic patients. This risk score has been identified as an independent prognostic factor for sepsis prognosis. Our findings offer novel insights into the role of ET in sepsis and lay a solid foundation for future research.

## Data Availability

The raw data supporting the conclusions of this article will be made available by the authors, without undue reservation.
