# Supplementary material for: Identification and validation of an endotoxin tolerance–based prognostic model with therapeutic insights in sepsis
Source: Front Immunol. 2026 Jan 15;16:1707490. doi: 10.3389/fimmu.2025.1707490 (PMC12852034; doi:10.3389/fimmu.2025.1707490)
Supplement: Supplementary file 1 [file Table1.docx]

**Supplementary Table 1**

| GENE | Forward (5′→3′) | Reverse (5′→3′) |
| --- | --- | --- |
| IL4R | CGTGGTCAGTGCGGATAACTA | TGGTGTGAACTGTCAGGTTTC |
| ATM | ATCTGCTGCCGTCAACTAGAA | GATCTCGAATCAGGCGCTTAAA |
| CX3CR1 | ACTTTGAGTACGATGATTTGGCT | GGTAAATGTCGGTGACACTCTT |
| F5 | TCCAGGCCGAGAATACACCTA | CGATTTGCTTGTCAAACGTCTTC |
| ELANE | CTCGCGTGTCTTTTCCTCG | GCCGACATGACGAAGTTGG |
| ADRB2 | TTGCTGGCACCCAATAGAAGC | CAGACGCTCGAACTTGGCA |
| TLR5 | GCCGGTCCTGTGTTTGGAAT | GGTGAGGTTGCAGAAACGATAAA |
| FCGR1A | TGGCCTTGAGGTGTCATGC | GCAAGAGCAACTTTGTTTCACA |
| CARD16 | TGGGTGAAGGTACAATAAATGGC | AGCTCGGGTCTTATCCATAACT |
| CCL5 | CCAGCAGTCGTCTTTGTCAC | CTCTGGGTTGGCACACACTT |
| ACTB | ACTGTCGAGTCGCGTCC | CTGACCCATTCCCACCATCA |

**Supplementary Table S1. Primer sequences (5′→3′) used for quantitative RT–PCR.**
